# The influence of diabetes mellitus II on cognitive performance

**DOI:** 10.1590/S1980-57642012DN06020003

**Published:** 2012

**Authors:** Juliana Luchin Diniz Silva, Lucas Trindade Cantú Ribeiro, Nina Razzo Pereira dos Santos, Vanessa Cristina Almeida de Sousa Beserra, Yara Dadalti Fragoso

**Affiliations:** 1 Undergraduate Medical Students, Universidade Metropolitana de Santos, Santos SP, Brazil.; 2 MD, MSc, PhD, Head of the Department of Neurology, Universidade Metropolitana de Santos, Santos SP, Brazil.

**Keywords:** diabetes mellitus, cognition, figure recognition, verbal fluency, 10×36 test

## Abstract

**Background:**

The association between diabetes mellitus and cognitive dysfunction is becoming increasingly clear, rendering it necessary for physicians in charge of diabetic patients to have the means to assess cognitive performance. Simple tests that can be applied during routine consultations may be useful for monitoring cognitive function during the course of diabetes.

**Objective:**

The objective of the present study was to assess cognition in diabetes mellitus type II (DM-II) using simple tests that can be incorporated into routine medical practice.

**Methods:**

A cross-sectional study including healthy controls and DM-II patients was carried out between May and September 2011. Volunteers aged 60 years and over were assessed by means of figure recognition, verbal fluency and the 10×36 tests.

**Results:**

A group of 100 participants was divided into a subgroup of 50 DM-II patients and a subgroup of 50 healthy volunteers. No statistical difference regarding demographic characteristics was found between the two groups. Results on the 10×36 test showed significantly worse performance among DM-II patients (p<0.0001). Assessment of the DM-II subgroup in terms of disease duration showed statistically significant differences (p<0.001) on figure recognition and verbal fluency, with worse cognitive performance among individuals with longer disease duration, irrespective of gender or age.

**Conclusion:**

Figure recognition, verbal fluency and 10×36 tests are easy to apply and could be used in routine medical practice for the early detection of cognitive dysfunction among patients with DM-II.

## Abstract (pt)

**Introdução:**

A associação entre diabetes mellitus e disfunção cognitiva está se tornando clara, exigindo que o médico que atende o paciente diabético tenha condições de fazer uma avaliação cognitiva. Testes simples que podem ser aplicados durante consultas de rotina podem ser de importância para monitorar disfunção cognitiva durante o curso do diabetes.

**Objetivo:**

O objetivo do presente estudo foi a avaliação da cognição em diabetes mellitus tipo II (DM-II) usando testes simples que podem ser incorporados na prática médica diária.

**Métodos:**

Um estudo transversal foi realizado no período entre maio e setembro de 2011, incluindo controles saudáveis e pacientes com DM-II. Voluntários com 60 anos ou mais foram avaliados para Reconhecimento de Figuras, Fluência Verbal e Teste 10×36.

**Resultados:**

Um grupo de 100 participantes foi dividido em um subgrupo de 50 pacientes com DM-II e um subgrupo de 50 voluntários saudáveis. Não houve diferença estatística entre as características demográficas O teste 10×36 mostrou desempenho significativamente pior nos pacientes com DM-II (p<0.0001). Quando o subgrupo de DM-II foi avaliado com relação à duração da doença, houve diferença significativa (p<0.001) para o Reconhecimento de Figuras e para Fluência Verbal, com pior desempenho cognitivo para indivíduos com maior tempo de doença, independente do gênero e idade.

**Conclusão:**

Reconhecimento de Figuras, Fluência Verbal e Teste 10×36 são testes fáceis de aplicar que poderiam ser utilizados na prática médica diária para identificação precoce de disfunção cognitiva em pacientes com DM-II.

## INTRODUCTION

Diabetes mellitus (DM) is a chronic disease characterized by dysfunction of secretion and usage of insulin, leading to hyperglycemia. The type II form of DM (DM-II) predominantly shows resistance to insulin, typically associated with a relative decrease in its secretion, which ultimately leads to multiple organ damage.^1^

DM-II increases the risk of cognitive dysfunction,^2^ with resultant worse performance on neuropsychological screening tests, irrespective of age or DM type.^3-5^ The underlying mechanisms of cognitive impairment in DM remain under investigation, but it seems clear that vascular endothelial disease, glucose and insulin abnormalities, dyslipidemia, metabolic syndrome, hypertension, obesity and amyloid metabolism are all involved, both separately and in association.^6^ Microscopically, cognitive dysfunction may be the result of hippocampal injury, reduction in grey matter density or microvascular changes to white matter.^7^

Irrespective of the mechanism underlying this cognitive impairment, patients with DM-II also present a wide variety of comorbidities and associated diseases requiring extensive care. In fact, cognitive dysfunction might contribute to a worsening in the clinical condition of such patients, who may forget medications or become more isolated in their social lives. Therefore, assessing the cognitive condition of DM-II patients should form part of routine medical practice, and not rely upon highly specialized (and often difficult to obtain) neuropsychological tests.

The Mini-Mental State Examination, the best known test for assessing dementia, is of limited usefulness for screening the general population without dementia^8^ and typically takes over ten minutes to apply.^9^ Therefore, simpler, briefer and easy-to-apply tests may be more effective for evaluating DM-II patients in daily practice.

The aim of the present comparative study was to assess the importance and validity of a battery of simple tests, namely the figure recognition, verbal fluency and 10×36 tests, in a group of DM-II patients versus a control group.

## METHODS

The present study was approved by the Research Ethics Committee of the Universidade Metropolitana de Santos, SP, Brazil, under process number 019/11.

Individuals drawn from the general population (for example, shoppers frequenting a fruit and vegetable market) were invited to participate in the study as control subjects. Inclusion criteria for these control individuals was that they had never been diagnosed with Diabetes mellitus and had tested negative for this condition within the last three months. Patients attending Diabetes mellitus medical units were invited to participate in the study as patients. Only individuals aged over 18 years were invited to participate. All diabetic patients had confirmed diagnoses and were undergoing treatment. Blood glucose levels or use of medications were not criteria for inclusion or exclusion of patients. At time of study inclusion, patients and controls presented no dementia complaint or diagnosis.

Schooling was classified into the following levels = zero (<4 years' formal education); 1 (4-8 years of primary education); 2 (9-12 years of schooling); 3 (University degree); 4 (Postgraduate degree).

Medical students were trained to apply the three tests and participants were recruited in the cities of São Paulo (SP), Limeira (SP), Santos (SP), Rio Claro (SP), Santo André (SP) and Cabo Frio (RJ).

After giving their written consent to participate in this study, all individuals were asked to answer the Hospital Anxiety and Depression (HAD) questionnaire,^10^ in order to exclude cases of moderate to severe anxiety and/or depression that could influence the cognitive performance results. Data on gender and age, medical history, body mass index and disease duration were recorded for the DM-II subgroup. Mean time since patient DM-II diagnosis was 86.0 months ± 90.7.

The battery of tests was carried out in a calm and quiet environment; all participants had had a full night's sleep and were not fasting. Participants were not undergoing chronic treatment with drugs affecting the central nervous system and, except for some previous histories of occasional primary headaches, did not suffer from any neurological or psychiatric disease.

Figure recognition was tested as recommended by Nitrini et al.^11^ Briefly, a sheet of paper with drawings of ten concrete figures was presented to the individual, who named each of them. Incidental memory was tested by asking the participant to recall the figures. The sheet was then presented again and the figures were named again. Immediate memory was then tested by asking the participant to recall the figures once again. The sheet of paper was re-presented and the participant was made aware that subsequent recall would be elicited after a short period of time, in order to test learning ability.

Verbal fluency was tested by asking participants to name as many animals as they could think of in one minute. It was explained that different genders of the same animal did not count as a correct answer. Results were adjusted according to schooling, as recommended by Brucki et al.^12^

Subsequently, the 10×36 test^13^ was applied. In this test analyzing visual-spatial memory, a table with 36 squares containing 10 random circle marks was presented to the individual for one minute. After this period, the table was then presented blank and the subject was asked to draw in the circles shown in the original table. The test has been routinely used with patients attending neurological outpatient services in the city of Santos, SP.^14^

After this third test, participants were asked to evoke the ten figures again. Another sheet of paper containing 20 figures was then shown, and participants had to point out which of these figures were part of the original ten-figure test.

After correcting for schooling, the results were organized for continuous data statistical assessment. For scoring, each value on the figure recognition (five in total) was summed to give a total number of correct answers, the final number of animals named was used for the verbal fluency result, and the number of correct circles marked on the 10×36 test was used to calculate final score on this test.

Statistical analysis included Student's t-test and Pearson's correlation coefficient, employing two-tailed p values. Values were considered statistically significant when p<0.05.

## RESULTS

Of the initial group of 104 volunteers, four were excluded (one case of depression and three cases of neurological disease). The remaining 100 participants were divided into a subgroup of healthy controls and a subgroup of DM-II patients. The demographic data did not differ between the two groups, except for body mass index, which was significantly higher in DM-II patients (p=0.02). All the demographic data are given in Table 1, together with a summary of results. For both groups, a negative correlation was found between age and performance on the figure recognition test: the older the patient, the worse the performance (r= –0.32; p=0.02). There was no correlation regarding age and performance on the verbal fluency test (r=0.46; p=0.16) or the 10×36 test (r=0.02; p=0.89). However, a positive correlation was detected between schooling level and performance on both figure recognition (r=0.30; p=0.03) and verbal fluency (r=0.38; p<0.01) tests. No statistically significant correlation between schooling and performance on the 10×36 test (r=0.24; p=0.08) was evident.

**Table 1:** Demographic data on patients with DM-II and control subjects

Total (n=100)	Control Group (n=50)	DM-II Group (n=50)
Females (n/%)	40/80%	33/66%
Males (n/%)	10/20%	17/34%
Age (years) mean±SD	70.8±7.1	70.1±6.8
Schooling (level)	1.8±1.4	2.2±1.1
Body mass index	1.8±0.9	2.3±1.1*
**Performance on tests**		
Figure Recognition	50.0±5.2	50.7±4.9
Verbal Fluency	15.7±5.6	16.0±4.5
10×36 test	4.2±2.0	2.3±1.1**

Schooling was classified into levels: zero (<4 years' formal education); 1 (4-8 years of primary education); 2 (9-12 years of schooling); 3 (University degree); 4 (Postgraduate degree). Body mass index was significantly higher in DM-II patients (*p=0.01). All other values showed no significant difference between the two groups (p>0.1). Mean values (±SD) for scores on Figure Recognition, Verbal Fluency and 10×36 tests showed that performance was significantly worse on the 10×36 test (**p<0.0001) in DM-II patients. All other values showed no significant difference between the two groups (p>0.1).

While no significant differences were observed between DM-II patients and controls on figure recognition and verbal fluency tests, a highly significant difference (p<0.0001) between these two subgroups was seen on the 10×36 test. Disease duration had no influence on 10×36 test performance, since no significant differences in results were observed among patients with different disease durations (from less than 60 months to over 240 months).

**Figure 2:**
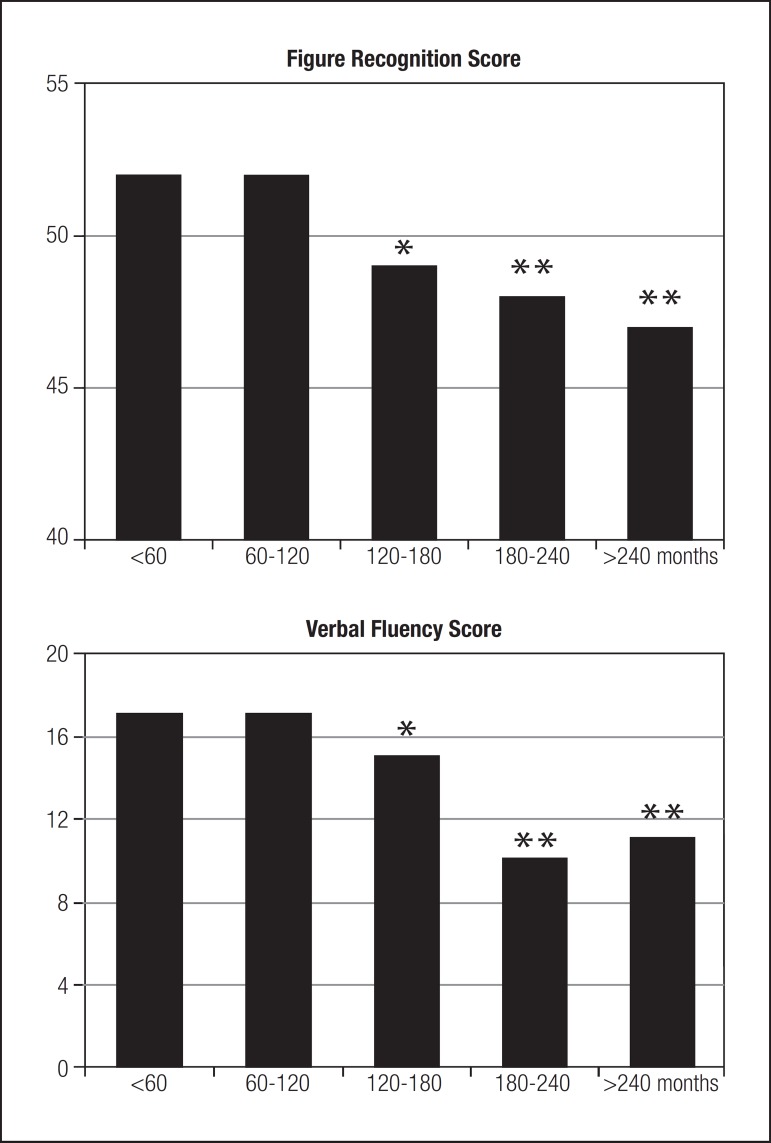
Influence of disease duration on Figure Recognition and Verbal Fluency test performance in DM-II patients. After 120 months of disease, significantly lower scores were achieved for both tests (*p<0.01). Scores were lower still after 180 and 240 months of disease duration (**p<0.0001from baseline).

A significant difference in patient performance on figure recognition and verbal fluency tests for disease duration was observed. A threshold of around 120 months of DM-II duration was found, after which average scores on both tests showed a significant decrease (p<0.001). Regarding figure recognition, results were worse after a further 120 months' follow-up (p<0.01in relation to value at 120 months, and p<0.0001in relation to disease duration of less than 60 months). The 10×36 test results appeared to be unaltered by disease duration (p>0.1), but were impacted by the presence of Diabetes (p<0.0001). These results are given in Figure 2 and Table 1.

In summary, both figure recognition (slow to apply) and verbal fluency (rapid to apply) tests proved to be reliable instruments for detecting cognitive impairment over the course of the disease, while the 10×36 test was more useful to screen for cognitive deficits in DM-II patients versus controls.

## DISCUSSION

Although another Brazilian study on cognitive decline and DM-II has recently been published by Alencar et al.,^15^ these authors employed the Mini-Mental State Examination, which takes longer to apply during a routine medical consultation (average of 16 minutes). In their study, Alencar et al. reported that the Mini-Mental State Examination required controlling for age, gender, schooling, hypertension and dyslipidemia. Such corrections may be difficult to achieve during regular consultations by non-specialists.

In the present study, simpler tests were used that take up less time in the medical consultation, which is invariably too short. The average time taken for the figure recognition assessment and the 10×36 test is less than four minutes each. Verbal fluency takes around one minute. If further studies confirm that both figure recognition and verbal fluency indeed evaluate only cognitive impairment over the course of disease duration, then one of these two tests could be selected for application in the same battery as the 10×36 test. Within five minutes, valuable data on patients' cognitive performance could be obtained without apparatus or specific training for applying these tests.

Patients with DM-II are at higher risk of developing dementia^16^ for reasons of neurodegeneration and/or microvascular changes,^17^ where both conditions may lower the threshold for more severe cognitive impairment. The recent methodologically sound study of Xu et al.^18^ showed that DM-II substantially accelerates progression from mild cognitive impairment to dementia in older patients. Earlier onset, longer duration and poor glycemia control are all associated with cognitive dysfunction in DM-II.^19^ The possibility of performing simple tests during regular consultations may prove important for the early detection and treatment of cognitive impairment secondary to DM-II.

The authors are aware that this study has inherent limitations due to its small sample size and the fact that no specific comparisons with the MMSE can be drawn from the present work.

To conclude, figure recognition, verbal fluency and the 10×36 tests are all short, simple and easy-to-apply instruments that may be included as part of routine medical consultations for DM-II patients. These patients are at high risk of developing cognitive impairment and dementia, thus adding to the already immense burden of DM-II.

## Figures and Tables

**Figure 1:**
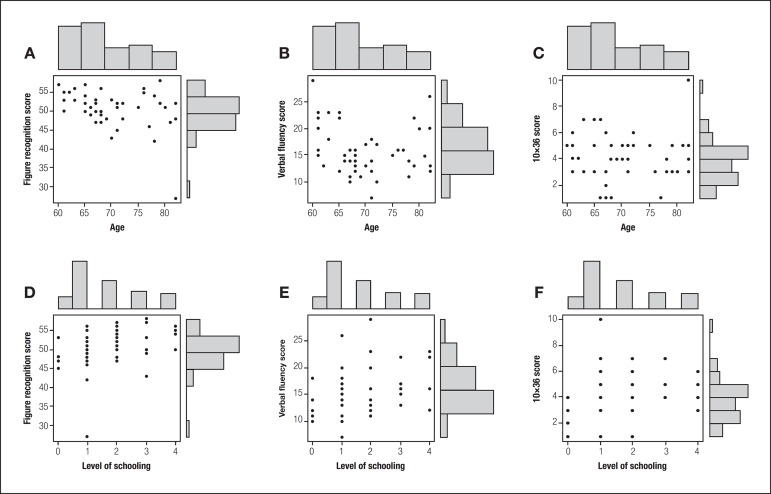
Pearson's correlation between age and schooling for the three tests. A significant negative correlation between age and figure recognition performance was noted [A]. No age effect on verbal fluency [B] or 10×36 [C] test performance was evident. Schooling was positively correlated with figure recognition [D]; no correlation between verbal fluency [E] and 10×36 [F] test performance was evident. [A] age × figure recognition; p=0.02; [B] age × verbal fluency; p=0.16; [C] age × 10×36; p=0.89; [D] schooling × figure recognition; p=0.03; [E] schooling × verbal fluency; p<0.0; [F] schooling × 10×36; p=0.08.
